# Patient Involvement in Safe Delivery: A Qualitative Study

**DOI:** 10.5539/gjhs.v8n6p33

**Published:** 2015-09-28

**Authors:** Forozun Olfati, Saeid Asefzadeh, Nasrin Changizi, Afsaneh Keramat, Masud Yunesian

**Affiliations:** 1 Student Research Committee, School of Nursing and Midwifery, Shahroud University of Medical Sciences, Shahroud, I. R. Iran; 2 Department of health, Qazvin University of Medical Sciences, Qazvin, I. R. Iran; 3 Center for maternal, fetal and Neonatal Research, Tehran University of Medical Sciences, Tehran, I. R. Iran; 4 Institute for Environmental Research, School of Public Health, Tehran University of Medical Sciences, Tehran, I. R. Iran; 5 Schools of Nursing and Midwifery, Shahroud University of Medical Sciences, Shahroud, I. R. Iran

**Keywords:** safe delivery, patient involvement, qualitative research

## Abstract

**Introduction:**

Patient involvement in safe delivery planning is considered important yet not widely practiced. The present study aimed at identifythe factors that affect patient involvementin safe delivery, as recommended by parturient women.

**Methods:**

This study was part of a qualitative research conducted by content analysis method and purposive sampling in 2013. The data were collected through 63 semi-structured interviews in4 hospitalsand analyzed using thematic content analysis. The participants in this research were women before discharge and after delivery. Findings were analyzed using Colaizzi’s method.

**Results:**

Four categories of factors that could affect patient involvement in safe delivery emerged from our analysis: patient-related (true and false beliefs, literacy, privacy, respect for patient), illness-related (pain, type of delivery, patient safety incidents), health care professional-relatedand task-related factors (behavior, monitoring &training), health care setting-related (financial aspects, facilities).

**Conclusion:**

More research is needed to explore the factors affecting the participation of mothers. It is therefore, recommended to: 1) take notice of mother education, their husbands, midwives and specialists; 2) provide pregnant women with insurance coverage from the outset of pregnancy, especially during prenatal period; 3) form a labor pain committee consisting of midwives, obstetricians, and anesthesiologists in order to identify the preferred painless labor methods based on the existing facilities and conditions, 4) carry out research on observing patients’ privacy and dignity; 5) pay more attention on the factors affecting cesarean.

## 1. Background

Mother health is of utmost importance as it is one of the eight major goals set for the millennium development (Sachs & McArthur, 2005). Nowadays, besides the indicators that show the average level of community health considering the components that indicate quality among population groups play an important role (Childbirth).

In spite of so many technological achievements of the 21^st^ century, about 44 million pregnant women in developing countries do not have access to prenatal care services. A nnually an average of 200,000 Asian women die because of the complications of pregnancy and delivery. Every year an estimated 5 million neonates die due to insufficient prenatal and delivery cares is (Organization, 2010).

The excessive number of demands for cesarean section is major concern for reproductive health experts. Unofficial statistics have reported higher percentages (80%); while the World Health Organization’s has suggested the figure is 10% - 15% (Education, 2009). Corresponding to death of each mother, 30 mothers suffer from diseases, injuries and permanent disabilities caused by complications during pregnancy and childbirth.

A quarter of women in developing countries, suffer from acute or chronic pregnancy-related diseases. The exact number of these cases is neither available in developed countries nor in developing countries (Donnay, 2000).

Safe delivery is a delivery assisted by educated and skillful individuals in a proper environment which is accessible at an affordable cost and within a short time. It is performed at the highest level of standard and through a proper method, and the result will be a healthy neonate and a healthy mother (Abou Zahr, 2003) (Childbirth).

In a study in rural and urban parts of Iran, Moradi Lakeh et al. (2010), found that safe delivery indicators across the country were a significantly differentinthe status quo and the condition of a full compliance with the standards. They attributed such differences to socioeconomic factors (Moradi-Lakeh, Ramezani, & Naghavi, 2007).

Adhering to principles of clinical governance helps improve the quality of clinical services. England follows clinical governance principles in providing prenatal services and in its daily reporting system. Mothers’ mortality rate in England is 13 per 100000 labors. According to a survey conducted in 2007, 89% of women were satisfied with the delivery services they received (Arulkumaran, 2010).

In order to improve the quality of clinical services, clinical governance was officially introduced in Iran1n 2009. Clinical governance is a framework that considers each service providing organization responsible for making persistent improvement in the quality of its services. Patient involvement is one of the seven pillars of clinical governance (Hooshmand, Tourani, Ravaghi, & Ebrahimipour, 2014; Ravaghi et al., 2014).

Evidence suggests that mothers are more dissatisfied with cares during childbirth than prenatal cares. Knowledge of and participation in decision-making processes as well as effective communication with service providers have been shown effective in the overall women’s satisfaction who received care (Brown & Lumley, 1994).

Engaging users can be particularly difficult with regards to clinical decisions. Within the provision of maternity care, there has been a significant shift in high-resource countries to accommodate the wishes of the mother and her family regarding the mode and place of delivery. Further involvement of the mother in complex clinical decision making is still not a common practice (Bodenheimer, MacGregor, & Sharifi, 2005).

More qualitative research is required to explore the effect of women’s satisfaction, autonomy and gender role in the decision-making process. Antenatal care cannot be achieved adequately merely through establishing health centers; women’s overall (socioeconomic and political) status needs to be taken into account as well (Simkhada, Teijlingen, Porter, & Simkhada, 2008).

Pregnant women can play a crucial role in improving patient safety by becoming actively involved in the health care system. However, there is a paucity of empirical evidence on the extent to which patients take on such roles. In order to encourage patient participation in patient safety, it is primarily required to assess the full range of factors that may be implicated in such involvement.

This research aimed at surveying factors that could affect patient involvementin safe delivery, as recommended by parturient women. In their social interactions, people may perceive one certain phenomenon in different ways mainly because of their previous personal experiences. Considering the significance of safe delivery and due to the role it has concerning mothers’ health, a qualitative research seems necessary to find out about the mothers’ views in this regard.

## 2. Method

In this qualitative study, the qualitative content analysis methodology has been employed. Colaizzi model was used for content analysis (Speziale, Streubert, & Carpenter, 2011).

The participants in this research included mothers before discharge after delivery. Participants were selected based on their experiences and the research objectives. A total of 68 eligible participants were selected for interview, of which 5were left out due to lack of interest to continue cooperation.

Inclusion criteria included tendency to participate in the research. Exclusion criteria of this study were based on lack of tendency toward continuing the cooperation.

Data were collected from postpartum women familiar with safe delivery before discharge from a hospital using purposive sampling through maximum variation sampling. Theoretical sampling was used to determine the number of participants and sessions. Interviews continued until data saturation was achieved the answers became repetitive.

Following a goal-oriented sampling, we performed semi-structured interviews to extract the themes. Each session lasted 1 to 1.5 hours. The interview process lasted until data saturation. The interviews were held in four hospitals and three cities in Iran: Shahroud, Qazvin and Tehran.

The questions were developed by researcher. These questions which were concordant with the goal of the predesigned plan and were used to control the interview sessions, were as follow:

1) What is a safe delivery, in your opinion?
2) Which factors may encourage pregnant women to involve insafe delivery?

Interviewer had good communication skills. Prior to recording interviewees’ voices, their written consent and the research aims were explained. In addition, they were assured that their identity would remain confidential. All of the participants read and signed the conscious satisfaction form designed by the research team. The interviews were conducted face-to-face.

For data analysis, after each interview, the interview tape was transcribed and analyzed. Finally, the findings were compared to the researcher’s interpretations. This finally led to explanation detailed description of “safe delivery” as perceived by the mothers. Comparative analysis was performed on the data in order to extract primary codes. In the next stage, themes were organized based on their concept into categories. In this part, the primary codes were classified based on differences and similarities in abstract categories and key concept (Speziale et al., 2011). The continuous analysis of data began from the beginning of codification, and continued until the end of data collection. Two of the authors participated in data coding process.

The credibility was owed to the researcher’s sufficient experience, scientific knowledge and academic degree. An ongoing engagement was accomplished as a result of the researcher’s constant mental engagement with the data and this led to an increase in the depth and size of the data. Persistent observation was accomplished through data reading and analyzing for several times and through applying a combination of methods such as face to face observation and interview. The participators’ handwritten texts were studied to verify the credibility of the extracted themes. The researcher’s reports and notes were submitted to another expert so that the similarity among the findings could be approved by both experts. The MAXQDA10 software was employed to manage codes.

All Ethical issues (such as conflict of interest, misconduct, co-authorship, double submission, etc.) have been considered carefully. Ethical permission (No. 9227) for the study was obtained from Shahroud University of Medical Sciences on February 17, 2012.

## 3. Results

A total of 63 mothers (mean age = 28 years), literate and illiterate from both urban and rural regions, participated in the study. The participants included mothers before discharge after delivery. Four broad categories of factors likely to influence patient involvement in safe delivery emerged. The findings are presented in Table 1.

**Table 1:** Themes, major categories, subcategories and codes

Theme	Main category	Subcategory	Main codes
**Patient-related**	Knowledge ⁄ beliefs	True beliefs	-Delivery cares should be efficient to reduce the labor pain. - Both baby and mother should be healthy; baby and mother should not face problem. - During labor, patient should be cooperative; I know that I acted annoyingly, we should be cooperative. - For my next pregnancy I’d like to give birth naturally.

		False beliefs	-Delivery should be assisted by a specialist.

	Demographic features	Literacy	- Educated women aremore likelytohave acaesarean section.

	Emotions and coping style	Privacy	- During labor, anyone in the delivery unitcame by to examine. - It is not comforting that the specialist is a man.

		Respectfor patient	- I was satisfied with the prenatal care. -I didn’t like to get delivery care from medical students.

**Illness-related**	Stage ⁄ severity of illness	Pain	- I prefer cesarean because it is less painful, I suffered the pain but ultimately I underwent cesarean operation. - For delivery of my first child, I suffered less pain in a private hospital. - Examinations were painful and too many.

	Treatment & outcomes	Type of delivery	- Now the number of women who choose cesareansection is increasing. We do not know which delivery mode to choose. - Normal delivery cancausepelvicprolapse. - I wish I had cesarean section from the first; I’ll have cesarean section whenever it is needed.

	Prior experience:	Patient safety incidents	-During labor and before that Iwanted to know more. - Specialists did not provide the required instructions. - Though I delivered at a private hospital, I had catheter for 4 days which caused infection.

**Health care professional-related& Task-related**	Interactions with patients	Behavior	- Personnel should treat patients tactfully. - Personnel should behave more properly.

	HCP_s professional role	Monitoring&Training	- I was under control of both a specialist and a health center and both were the same. - It would be better if instructions were provided through media (Radio & TV). - Normal delivery is better but my physician warned me because I was too old and I had a narrow pelvis. - They should diagnose early so that patients can be provided with the required services sooner. - I was monitored by both my physician and the health center; yet the health center was more effective because of its instructions.

**Health care setting - related**	Health care setting	Financial aspects	- Private hospitals offer more facilities but they are expensive. - There are some private pregnancy and breastfeeding courses, but that I could not afford to attend them. - Generally the costs are high and we are not insured; we should pay a lot for each ultrasonography examination. - They should provide us with insurance coverage otherwise we cannot afford the costs.

		facilities	- Patients should get equal access to facilities. -Patients should refer to clinics and to centers providing prenatal cares in a timely manner. - I wish I got training in prenatal cares and knew more about delivery. - It’s so far away; There’s just one health center in our village; I had to come all the way to Shahroud to visit my physician. - When it snows, the roads are closed; since I had bleeding, they refused to hospitalize me even though I had travelled a long distance to the center. - I delivered at night, there was only one midwife to assist and she was too busy to handle all other patients. -Wedon’t know the centers that provide the instructions; specialists are reluctant to give information

### 3.1 Patients-Related

Mothers believed that they should act more cooperatively. One mother said, “We should be cooperative and attend the training courses. We should observe the instructions and orders.”

### 3.2 Illnesses-Related

One mother noted, “Now the number of women who consider cesarean delivery is increasing. We don’t know which delivery mode to choose. I myself prefer cesarean because it is less painful.”

### 3.3 Health Care & Professional-Related & Task-Related

One mother said, “Delivery should be carried out naturally; however, my physician said you’d better get a cesarean delivery because I was not young enough and my pelvis was too narrow.”

As for the role of information in a safe delivery, most of the mothers were not aware which centers to refer to as to obtain information. One said, “I did not know where to obtain the required information. Radio and television should provide more information.”

Participants believed the number of educated personnel, mainly midwives, was insufficient. One of the mothers said, “The number of nurses and midwives is not enough and they are too exhausted to serve the patients.”

### 3.4 Health Care Setting-Related

As for the facilities and equipment, mothers compared the facilities of private hospitals with those of public hospitals concluding that the facilities in private and public hospitalswere not different.

The participants believed that financial issues had been the major problem preventing physiciansfrom attending. One mother said, “I preferred to be visited by a specialist but I could not afford it because I had no insurance coverage.”

One mother noted, “All private hospitals do is charging patients money. They’re no different than public hospitals.”

One mother said, “People should get insurance coverage so that they can afford advanced delivery services.”

Mothers pointed out some major problems they faced: the long distance they had to travel to get to the medical centers, absence of insurance coverage, and their own insufficient knowledge about the proper mode of delivery. One mother said, “The number mothers undergo cesarean delivery is increasing but personally I don’t know which delivery mode is the best for me, vaginal delivery or a caesarean delivery.”

As for the role of education in a safe delivery, almost everyone placed great importance on education. One of the mothers said, “I was visited by both a physician and a midwifein the health center. However, the health center was better because of its educations.”

The participants believed that delivery pain should be controlled and that physicians should recommend the proper delivery mode for each individual.

## 4. Discussion

Four categories of factors emerged that could affect patient involvement in safe delivery: patient-related (True and false beliefs, Literacy, Privacy, Respect for patient), illness-related (Pain, Type of delivery, Patient safety incidents), health care professional-related and task-related factors (Behavior, Monitoring, & Training), health care setting-related (Financial aspects, Facilities).

Educated patients are more likely to obtain health information and process and understand it. Furthermore, this is effective for patients’ decision-making and their participation (Arora & McHorney, 2000).

Right of privacy is the major human right whichis strongly linked to human dignity and moral principles. Legally protected, its purpose is to elevate human’s personality and her spiritual and physical integrity. It is originated from human rights principles and is essential for everyone regardless of the situation. The significance of patients’ criminal protection is increasingly observed, by the pertinent authorities due to the specific mental and physical condition patients have and its effect on the therapeutic process. By the same token, patients’ privacy is categorized under different classes: physical, mental and informational (Khaleghi & Joodaki, 2011).

“Exploring the concept of patients’ right to privacy, Woogara noted: I argue that health professionals can violate patients’ privacy in a variety of ways. For example: the right to enjoy their property; the right to protect their medical and personal information as confidential; the right to expect treatment with dignity during intimate care; and the right to control their personal space and territory. Some preliminary evidence indicates that many health care practitioners, including nurses, are presently unaware of the articles of the Convention and the implications of the Human Rights Act 1998. In order to prevent litigation for breaches of patients’ privacy, it is advocated that universities and other educational institutions, the Government and NHS (National Health Service) trusts should help to produce a clear educational strategy and protocols so that students and practitioners are well informed in this field”(Woogara, 2001, p. 234).

Previous patient’s experience stimulates the patient’s negative emotional reactions and a sense of vulnerability and anxiety which increases the sense of vulnerability of the patient in future health problems (Davis, Jacklin, Sevdalis, & Vincent, 2007). Vague information, about the relationship between disease severity and priority for intervention is influential to just the effect of the illness severity on the patient’s participation in health and treatment problems. (Thorne & Paterson, 2001)

Typically, labor pain, makes a pregnant woman nervous. Sometimes it is the main consideration both for the woman and for her family (Wong, Perry, Hockenberry, Lowdermilk, & Wilson, 2005). Labor pain is acute and becomes intense within a short period of time. It is affected by a set of factors including physiological, mental, social, cultural and environmental (Caton et al., 2002). Dick Reid (2011) demonst rated that unknown phenomena such as delivery, causes muscular contraction and increases the intensity of delivery pain (Bennett, 2005).

Attending pre-pregnancy education classes is a good opportunity to help mothers effectively communicate with service providers. In these classes, mothers can reconcile decisions with hospital policies, procedures and protocols (Nolan, 2009).

The research conducted by Miquelutti et.al in 2013 in Brazilia province, revealed that women who participated in the Birth Preparation Program (BPP) reported self-control during labor and used non-pharmacological techniques to control pain and facilitate labor and expressed satisfaction with the birthing experience (Miquelutti, Cecatti, & Makuch, 2013).

On a collective level, prior experience of a patient safety incident can result in apatient becoming involved in patient safety issues for patients as a whole (Mansell, Poses, Kazis, & Duefield, 2000).

Health care policy-makers are expected to attend to the factors influencing women’s decision-making on the childbirth method to reduce the number of unnecessary caesarean sections.

In a research conducted by Abbaspoor et al. (2014) in Iran, a vicious cycle of causes and factors was found which determined the delivery mode chosen by mothers. Mother’s demand can be one of the reasons for prevalence of cesarean delivery. However, socio-cultural, religious and economic norms in the Iranian society playthe main role in the selection of the birth method by Iranian women (Abbaspoor, Moghaddam-Banaem, Ahmadi, & Kazemnejad, 2014).

As for the financial considerations, the research carried out by MoradiLakeh et al in 2010, in rural and urban areas of Iran, revealed, women of higher economic classes with higher educational degrees are more likely to give birth in a proper place and be assisted by educated staff. As for the mode of delivery, the number of normal vaginal deliveries is decreasing with the improvement of economic and educational status; cesarean operation is more common place infamilies with higher socioeconomic status (Moradi-Lakeh et al., 2007).

Women’s tendency toward cesarean in the absence of medical indications and supply-induced demand contribute to the rise of cesarean section rates (Penna & Arulkumaran, 2003). Research shows leading to increasing requests by women for cesarean section: Choice of a specific birth date, fear of pain, the wish to keep fit, and the belief that cesarean section is safer than vaginal delivery (Li, Wu, Wang, Xu, & Gao, 2006).

Health care providers’ attitude towards cesarean section is likely to have a significant impact on women’s perspectives because mothers consider them as the major information source. For health care providers, cesarean section is being performed at the controlled time and in a short term, and can avert prosecution arising from medical malpractice. Cesarean section is also more profitable than vaginal delivery (Long et al., 2012).

It has been reported that the way in which health care professionals^,^ interact with patients can affect patient participation in health care (Little et al., 2004). Conversely, participation can be decreased by health care professionals who are dismissive towards the patients^,^ concerns (Sainio, Lauri, & Eriksson, 2001).

As a major limitation of the study concerned, we were unable to include opinions of participants’ husbands. Husbands often have poor participation in safe delivery in Iran.

## 5. Conclusion

The potential for involving patients in maternity care is considerable but further research is needed to determine the influences on patient Participation constraints and possible risks.

It is therefore, recommended to: 1) Take notice of mother education and their husbands, midwives and specialists attitude; 2) Provide pregnant women with insurance coverage from the outset of pregnancy, especially during prenatal period; 3) Form a labor pain committee consisting of midwives, obstetricians, and anesthesiologists in order to identify the preferred painless labor methods based on the existing facilities and conditions, 4) Carry out research on observing patients’ privacy and dignity, 5)pay more attention on the factors affecting cesarean.
